# Supplementary material for: Vitamin D Modified DSS-Induced Colitis in Mice via STING Signaling Pathway
Source: Biology (Basel). 2025 Jun 18;14(6):715. doi: 10.3390/biology14060715 (PMC12190092; doi:10.3390/biology14060715)
Supplement: Supplementary file 1 [file biology-14-00715-s001.zip › Supplementary Materials S1: 16s-report of gut microbiota/Groups/diff_analysis/TukeyHSD/Tukey_GMM_rotate.pdf]

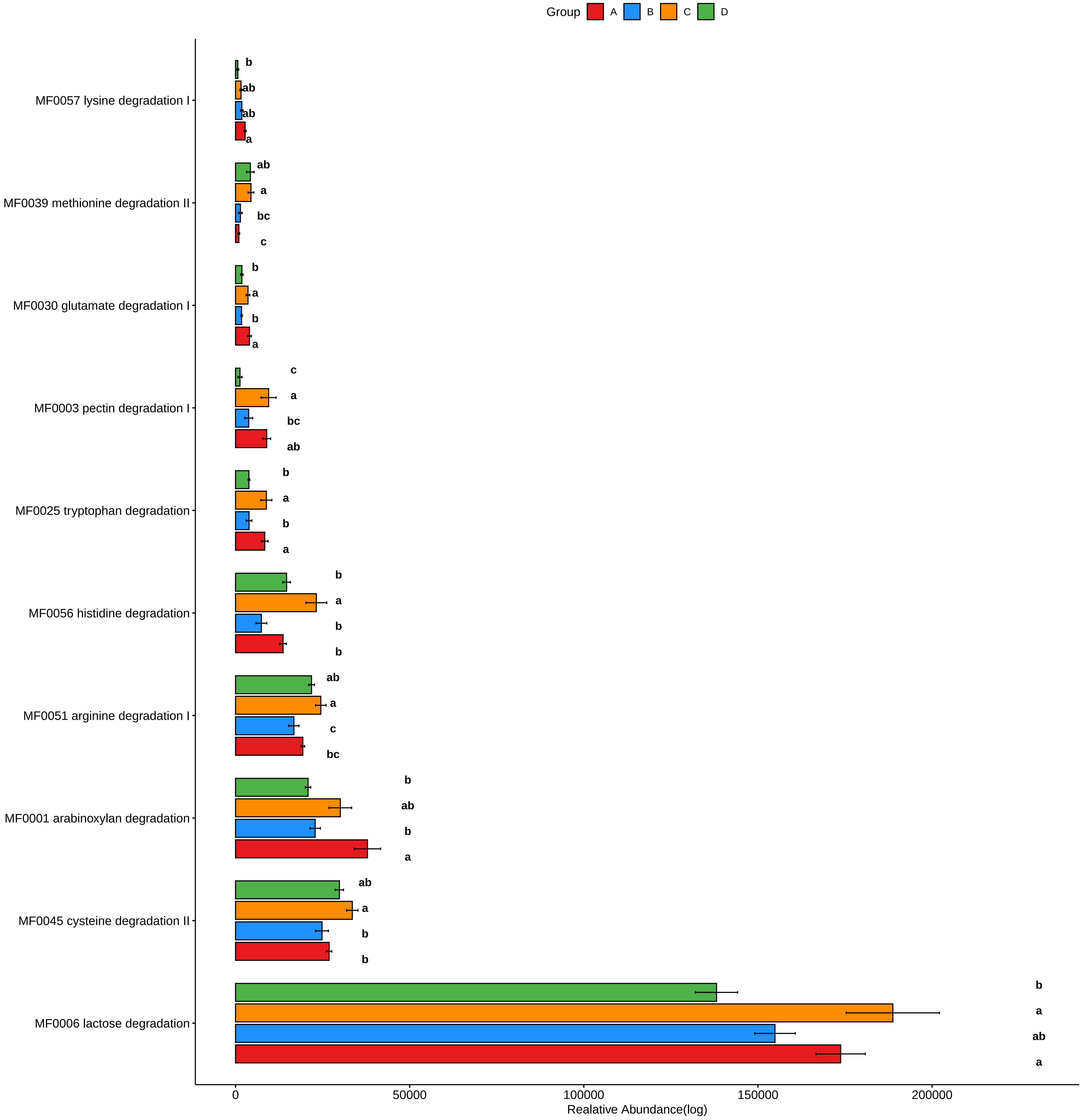

Group
A
B
C
D
MF0057 lysine degradation I
MF0039 methionine degradation II
MF0030 glutamate degradation I
MF0003 pectin degradation I
MF0025 tryptophan degradation
MF0056 histidine degradation
MF0051 arginine degradation I
MF0001 arabinoxylan degradation
MF0045 cysteine degradation II
MF0006 lactose degradation
b
ab
ab
a
ab
a
bc
c
b
a
b
a
c
a
bc
ab
b
a
b
a
b
a
b
b
ab
a
c
bc
b
ab
b
a
ab
a
b
b
b
a
ab
a
0
50000
100000
150000
200000
Realative Abundance(log)
